# Structure and Evolution of Ribosomal Genes of Insect Chromosomes

**DOI:** 10.3390/insects15080593

**Published:** 2024-08-04

**Authors:** Vladimir E. Gokhman, Valentina G. Kuznetsova

**Affiliations:** 1 Russian Entomological Society, Moscow 111024, Russia; 2 Department of Karyosystematics, Zoological Institute, Russian Academy of Sciences, St. Petersburg 199034, Russia

**Keywords:** AgNOR, chromomycin A_3_, FISH, Insecta, NORs, rDNA clusters

## Abstract

**Simple Summary:**

The class Insecta constitutes the largest group of terrestrial animals, with more than a million described species. As in all other animals, insect genomes contain clusters of specific genes, which are essential for producing and assembling ribosomes. These clusters harbor two types of repetitive DNA, i.e., 45S and 5S ribosomal DNA (rDNA). Currently, 45S and 5S rDNA clusters have been studied in about 1000 and 100 insect species, respectively. Although the number of species with known 45S rDNA clusters constitutes less than 0.1 percent of the described members of this enormous group, certain conclusions can already be drawn. Since haploid karyotypes with single 45S and 5S rDNA clusters predominate in both basal and derived insect groups, this character state is apparently ancestral for the class Insecta in general. Nevertheless, the number, chromosomal location, and other characteristics of both 45S and 5S rDNA sites substantially vary across different species, and sometimes even within the same species. There are several main factors and mechanisms that either maintain these parameters or alter them on the short-term and/or long-term scale.

**Abstract:**

Currently, clusters of 45S and 5S ribosomal DNA (rDNA) have been studied in about 1000 and 100 species of the class Insecta, respectively. Although the number of insect species with known 45S rDNA clusters (also referred to as nucleolus-organizing regions, or NORs) constitutes less than 0.1 percent of the described members of this enormous group, certain conclusions can already be drawn. Since haploid karyotypes with single 45S and 5S rDNA clusters predominate in both basal and derived insect groups, this character state is apparently ancestral for the class Insecta in general. Nevertheless, the number, chromosomal location, and other characteristics of both 45S and 5S rDNA sites substantially vary across different species, and sometimes even within the same species. There are several main factors and molecular mechanisms that either maintain these parameters or alter them on the short-term and/or long-term scale. Chromosome structure (i.e., monocentric vs. holokinetic chromosomes), excessive numbers of rRNA gene copies per cluster, interactions with transposable elements, pseudogenization, and meiotic recombination are perhaps the most important among them.

## 1. Introduction

Insects, i.e., members of the class Insecta, constitute the largest group of terrestrial animals, with more than a million described species [1,2]. The morphological and genetic structure of insects at various levels of organization is highly diverse, but it is often based on a combination of a limited number of elements/characteristics (see, e.g., [3]). Among these characteristics, there are many chromosomal features [4,5,6,7,8,9], including the number and structure of ribosomal genes [10,11,12]. These genes are essential for producing and assembling ribosomes [11,12,13,14]. Although some general characteristics of ribosomal DNA (rDNA) clusters in the phylum Arthropoda, which harbors a few large taxa, including insects, were reviewed in [15,16], together with certain similar reviews at the level of some insect orders [17,18,19] and lower taxa [20,21,22,23], these chromosomal regions were never reviewed in detail across the whole class Insecta. We recently reviewed and summarized all available information on the main structural, functional, and evolutionary features of rDNA clusters in insects. The results of this work are given below.

## 2. General Structure of Ribosomal Genes in Insects

As in other animals, ribosomal RNA (rRNA) genes in insects are represented by the larger and smaller transcriptional units that code 45S and 5S rRNA [10,11,15,24]. Clusters of 45S rRNA genes, the positions of which on metaphase chromosomes often can be recognized by so-called secondary constrictions, define the location of the nucleoli within insect cells, and hence are called nucleolus-organizing regions (NORs), whereas 5S rRNA ones are usually extranucleolar [4,10,11,13,14] (also see below). Each 45S rDNA unit contains three rRNA-coding sequences, 18S, 5.8S, and 28S, which are separated by internal transcribed spacers (ITS1 and ITS2) and two external transcribed spacers (5′ETS and 3′ETS) that flank these sequences [11,14,24,25,26]. Numerous 45S rDNA sequences are organized in tandem repeats, which are arranged in a head-to-tail fashion [10,13]. In insects, the number of copies of 45S rDNA per haploid genome can vary from 40–45 in some species of Chironomidae and Sciaridae (Diptera) to 240–280 in *Bombyx mori* (Linnaeus) (Lepidoptera, Bombycidae) and certain *Drosophila* Fallén species (Diptera, Drosophilidae) [27]. The 45S rDNA sequences are arranged in clusters and separated by external non-transcribed or intergenic spacers (ENSs or IGSs) [24,28]. Multiple copies of 5S rDNA sequences are also separated by ENSs (often referred to as non-transcribed spacers, or NTSs) and usually arranged in clusters [11,15,29,30], but see [31]. In the order Lepidoptera and the genus *Drosophila*, the 5S rDNA copy number per haploid genome varies from 13 to 264 and from 100 to 320, respectively [27,32].

## 3. Number and Position of Ribosomal Genes in Insects

The locations of 45S and 5S rRNA genes on insect chromosomes usually do not coincide [16,27,33], although clusters of these genes can be found on the same chromosome, e.g., [34]. However, at least three cases of co-localization of these genes are known, including the grasshopper *Rhammatocerus brasiliensis* (Bruner), the desert locust *Schistocerca gregaria* Forsskål (Orthoptera, Acrididae), as well as the darkling beetle *Lagria villosa* (Fabricius) (Coleoptera, Tenebrionidae) [35,36,37].

Haploid insect genomes often contain single clusters of both 45S and 5S rDNA, but their numbers are sometimes substantially higher; the latter seems more characteristic of 5S rDNA clusters [16,21,38] (also see below). In fact, multiple rDNA clusters have been reported in a number of insects, e.g., in ants of the genus *Myrmecia* Fabricius (Hymenoptera, Formicidae), the mealybug *Planococcus citri* (Risso) (Hemiptera, Pseudococcidae), the true bug *Chariesterus armatus* (Thunberg) (Hemiptera, Coreidae), some beetles, moths, and butterflies, and in the South African heelwalker *Karoophasma biedouwense* Klass et al. (Austrophasmatidae) from the recently discovered order Mantophasmatodea [31,32,39,40,41,42,43]. In insects, rDNA clusters are often situated on autosomes ([41,44,45,46,47,48,49] etc.), although they can also be located on sex chromosomes ([20,41,46,47,48,50,51,52,53,54,55] etc.), on B chromosomes [56,57,58,59,60], or on microchromosomes (m-chromosomes) [47,50,61,62,63,64]. Moreover, in many of the abovementioned species, 45S and 5S rDNA clusters are located on the chromosomes of two or more types simultaneously, e.g., on both autosomes and sex chromosomes. In addition, some insects show extraordinary diversity in rDNA location [22,62,65,66,67,68].

In insects, NORs are usually subterminal or, less frequently, pericentromeric ([16,18,19,69] etc.). In some species, these sites are distributed along the entire arm [21], sometimes occupying most parts of the chromosome [70]. In certain insects with holokinetic chromosomes (see below), 45S rDNA can also be detected almost along the half of a particular chromosome [32]. In contrast to this pattern, 5S rDNA clusters are most often pericentromeric but sometimes subterminal; nevertheless, clusters of both types can be interstitial [15]. However, the evolutionary mechanisms that apparently define this uneven distribution of 45S and 5S rDNA clusters in insects currently remain unclear, although they are widely discussed in the literature (see below).

It is also noteworthy that, apart from monocentric chromosomes, i.e., those with localized centromeres, holokinetic (=holocentric) chromosomes lack definite centromeric regions, and therefore rDNA clusters on these chromosomes cannot occupy pericentromeric positions. In turn, this effectively means that only subterminal and interstitial locations of rRNA genes are possible in holokinetic chromosomes. Taken together, the available evidence suggests that insects with holokinetic chromosomes have a predominantly subterminal position of ribosomal genes (see below). Furthermore, it is suggested [71] that rearrangements leading to the origin of interstitial rDNA sites are less constrained in organisms with monocentric chromosomes than with holokinetic ones. Although this hypothesis is based on cytogenetic information on both angiosperm and gymnosperm plants, data on the large insect taxa are apparently consistent.

The number and location of 45S and 5S rDNA clusters present in the genome of many organisms, including insects, often vary between species ([22,39,44,46,54,66,69,72,73,74,75,76,77,78,79,80,81,82,83,84,85,86,87,88] etc.). Moreover, these differences can delimit closely related species. For instance, the ants *Camponotus renggeri* Emery and *C. rufipes* (Fabricius), which share the same chromosome number, 2*n* = 40, and were subject to synonymization discussion in the past, nevertheless, have four and two 18S rDNA clusters, respectively [89]. In addition, the aforementioned parameters as well as the content of 45S and 5S rDNA clusters can vary within species, and even between cells of the same individual [10,66,69,72,90,91,92,93,94]. For example, in *Anurogryllus* sp. (Orthoptera, Gryllidae), this variation involves a pair of acrocentrics as well as one of the two metacentric chromosomes. In the latter case, an additional NOR apparently originated via translocation of the corresponding chromosome segment [95]. Furthermore, the diploid karyotype of a certain strain of *Aedes aegypti* (Linnaeus) (Diptera, Culicidae) carries an additional unpaired 18S rDNA site [96], in contrast to other strains of the same species. On the other hand, size heteromorphism of rDNA clusters can be observed among both chromosomally monomorphic and polymorphic populations [21,53]. Finally, expression of rDNA clusters is also subject to intraspecific variation [97,98]. For instance, the total nucleolar volume in males of the lamenting grasshopper *Eyprepocnemis plorans* (Charpentier) (Orthoptera, Acrididae), many of which carry an additional 45S rDNA cluster on the B chromosome, remains more or less constant [58]. This apparently means that variations in the expression level of the additional NOR maintains approximately equal nucleolar volume, regardless of the level of activity of this 45S rDNA cluster.

## 4. Functioning of Ribosomal Genes

Transcription of 45S and 5S rRNA genes is performed by DNA-dependent RNA polymerases I and III, respectively [10,24,30,99]. When transcribed, 45S rDNA produces a pre-rRNA that undergoes modification to remove ITSs and ETSs as well as to yield mature rRNAs (see above). As for ENSs, they harbor gene promoters and regulatory elements that control pre-rRNA synthesis [10]. In all eukaryotes, the rDNA copy number is much higher than actually required to produce the amount of rRNA transcripts necessary for ribosome biogenesis, since only about 50% of rRNA genes are transcribed [14]. It is therefore not surprising that the number of rDNA copies is subject to enormous variation, both inter- and intraspecific (see above). Specifically, this number can vary from 30 to 30,000 copies per genome [100], although this range is usually restricted to 100–1000 copies [101]. On the other hand, it turns out that the so-called redundant number of copies of rRNA genes maintains genome integrity by facilitating recombination repair [102]. In addition, *Drosophila melanogaster* Meigen exhibits cyclic changes in the number of these copies during ontogenesis, which can apparently be applied to other insects as well [103]. In this species, there is also an interesting example of allelic inactivation of certain rDNA clusters. Specifically, as noted above, these sites are located on both the X and Y chromosomes of *D. melanogaster* [51]; however, the entire cluster on the X chromosome is active in the female but totally silenced in the male, in which the cluster on the Y chromosome is expressed [103].

Nucleoli assemble around NORs at the end of mitosis [13]. In the oocytes of some insects, such as *Acheta domesticus* Linnaeus (Orthoptera, Gryllidae) and *Dytiscus marginalis* (Linnaeus) (Coleoptera, Dytiscidae), rDNA is amplified as short circular units that are temporarily associated with the nucleolus, and then are dissociated and dispersed as actively transcribed units at the diplotene stage [104]. In addition, DNA sequences from both larger and smaller transcriptional sites of rDNA sites can also form circular units in *D. melanogaster*, although their precise function is currently unclear [105,106].

Since rDNA clusters experience both intensive transcription and replication, these processes often collide, making the aforementioned chromosome segments extremely unstable [11,24]. This can lead to either duplications or deletions of rDNA, which, in turn, can cause specific phenotypic changes. For example, the characteristic appearance of the so-called *Bobbed* mutants in *D. melanogaster* is attributed to huge deletions that affect more than half of the wild-type 45S rDNA [27,107]. In addition, rDNA in insects, and its intergenic spacers in particular, is often associated with transposons and their remnants due to imprecise excision [108,109,110], and apparently can easily spread across different genomes via transposition [17,65,82,111,112]. In turn, this can lead to dramatic changes in both the location and copy number of rRNA genes (see below). The instability of rDNA clusters can also be judged from the apparent fragility of these sites in many organisms, including insects [113,114,115]. However, substantial decondensation of chromatin within rDNA sites does not necessarily lead to chromosomal instability (see, e.g., [116]).

## 5. Techniques for Identifying Ribosomal Genes

On conventionally stained chromosomes, NORs (i.e., 45S rDNA clusters) are usually recognized as achromatic gaps, often referred to as secondary constrictions [10,12] (also see above), although they are not always visible. In such cases, NORs can be visualized using so-called physical mapping. Historically, AgNOR-banding, which involves staining NOR-produced proteins with silver ions, was the first method used for such mapping [12,117]. This technique is still relevant (see, e.g., [49,53]), but now it is being actively displaced by fluorescence in situ hybridization of DNA, or FISH [33,118]. Indeed, with increasing numbers of NORs, not all of them remain active, and, consequently, are not visualized by AgNOR-banding [16,17,119]. For example, in some members of Pamphagidae (Orthoptera), all NORs are usually AgNOR-negative, although they are easily detected by FISH [120]. On the contrary, some NORs do form nucleoli, but they are not visualized by FISH [17,92]. In addition, FISH provides the only effective technique of physical mapping of 5S rDNA clusters ([31,32,35,36,37,38] etc.).

Discrepancies between results obtained by different techniques, such as silver impregnation and FISH, are often observed in insects, especially in the order Orthoptera. For example, [119] suggested that the high amount of inactive rDNA in the ladder grasshopper *Stauroderus scalaris* (Fischer von Waldheim) and, consequently, a number of silent rDNA sites in this species, could be explained by competition of these genes for transcription factors, and, therefore, the only active rDNA cluster (perhaps the largest one) could provide the cells with the sufficient amount of material necessary for ribosome production (see above). In addition, the position of rDNA clusters on chromosomes can also affect their transcription rates [121]. On the other hand, it is proposed in [17] that inactive or silent rDNA loci are those undergoing the process of elimination. In contrast to these loci, cryptic NORs, i.e., those that form small nucleoli but cannot be detected by FISH, are believed to be nascent rDNA clusters. It is also postulated in [17] that the abundance of both cryptic and silent rDNA loci in Orthoptera denotes that rDNA sequences spread across the genomes of these insects via transposition [111]. After being transposed, these sequences can either be amplified, giving rise to new NORs, or eliminated. Finally, most experts believe that AgNOR-banding sometimes provides non-specific and/or unreliable results (see, e.g., [21] and references therein).

As a rule, NORs are enriched with GC base pairs, which is likely due to GC-biased gene conversion [122] (also see below). Consequently, they are usually stained with chromomycin A_3_ (CMA_3_), which selectively binds to these nucleotides. Nevertheless, karyotypes with multiple CMA_3_-positive regions that often do not coincide with NORs are known among insects [123,124,125]. On the other hand, non-CMA_3_-positive rDNA sites are also detected in some cases ([96,126] etc.). Nevertheless, frequent association of NORs with heterochromatic regions [24,127] can also help to identify true rDNA clusters, especially in dubious cases.

## 6. Evolution of Ribosomal Genes

There are two apparently competing hypotheses on the general features of the existing distribution and evolution of 45S rDNA clusters in insects, which are discussed in some contemporary reviews on this subject (see, e.g., [23]). The first hypothesis postulates that subterminal rDNA sites are prone to spreading across the genome as a result of ectopic recombination in meiosis via terminal association and subsequent recombination between rDNA and constitutive heterochromatin arrays. On the contrary, intrachromosomal clusters of this kind, which are unlikely to multiply through the abovementioned mechanism, usually retain their lower numbers, and therefore often remain single within the haploid genome [12]. However, according to another hypothesis, the multiplication of rDNA clusters occurs mainly via chromosomal fission, thus establishing a correlation between the abundance of these clusters and the chromosome number [39]. Although the actual patterns observed in nature often defy unequivocal interpretation, we believe that these hypotheses are complementary rather than mutually exclusive. Specifically, the latter hypothesis seems plausible, at least in those cases when the number of rDNA sites correlates with the chromosome number, e.g., in the ant genus *Myrmecia* as well as in some other members of the order Hymenoptera [19,44]. However, these patterns are obviously not ubiquitous for the whole class Insecta, since, for instance, the abovementioned correlation is not observed in Lepidoptera [18,128] and Coleoptera [69]. This means that the latter hypothesis might lack generality, in contrast to the former one, which explains the spreading of rDNA clusters by processes occurring during meiotic recombination [12].

A correlation between the number of 45S rDNA sites and the amount of nuclear DNA also exists, at least for some groups of insects (e.g., Orthoptera if compared to other orders of Insecta; see below), because, for example, a similar correlation between the amount of nuclear DNA and the copy number of 45S rRNA genes is characteristic of eukaryotes in general [100]. Moreover, pseudogenization of ribosomal genes appears to be observed in some members of Diptera, in which 45S rDNA sequences contain characteristic insertions [27]. Finally, NOR-like sequences can spread over the whole genome in some cases ([31,129]).

Since distribution patterns of 5S rDNA across insect karyotypes are studied much more fragmentarily than those of 45S rDNA, our knowledge of the evolutionary pathways of 5S rDNA clusters is restricted [31,34,38,41,69,130,131,132]. Moreover, certain evidence suggests that factors governing the evolution of 45S and 5S rDNA can substantially differ [31,69]. For example, clusters of histone genes and 5S rDNA are often co-localized ([69,130,131,132] etc.), and therefore the number of these rDNA sites can be restricted by selection against the spreading of histone gene clusters.

As discussed above, the number and position of rDNA clusters on chromosomes as well as the copy number of these genes can substantially vary among related species or at the intraspecific level [44,47,66,73,74,133,134]. Multiple sequences of 45S and 5S rDNA generally retain their within-species uniformity and are generally believed to be the most conserved genes in eukaryotes (but see, e.g., [26,135,136,137]). Nevertheless, for instance, ITS1 and ITS2 are subject to considerable variation due to relatively weak selection pressure [11,138]. This variation is mostly interspecific (but see [98]), and ITS sequences are therefore widely used in molecular phylogenetic studies (e.g., [139]). The aforementioned pattern, termed “concerted evolution” [15,26,28,140,141,142], is likely explained by a number of molecular processes, among which homologous recombination, including unequal crossover and gene conversion, plays a leading role [11,122,138]. In addition, concerted evolution of both 45S and 5S rDNA can interact with the so-called birth-and-death mechanism, which may lead to the spreading of these sequences across the genome and/or their pseudogenization [31,38,129].

## 7. Specific Features of Ribosomal Genes in Various Orders of Insecta

The class Insecta contains about two dozen orders of winged insects (Pterygota) as well as two orders of primarily wingless insects, i.e., Archaeognatha and Zygentoma (Apterygota). However, information on the physical mapping of rDNA clusters in these insects is currently lacking.

**Palaeoptera (basal winged insects)** includes two orders, Ephemeroptera and Odonata. Nothing is known about the number and position of 45S rDNA sites in the former order. On the contrary, this information became available during the last decade for more than 20 Odonata species [64,118,143,144], which possess holokinetic chromosomes [145]. These species include 15 members of the families Aeshnidae, Corduliidae, and Libellulidae, as well as seven members of Calopterygidae and Coenagrionidae that belong to the suborders Anisoptera and Zygoptera, or dragonflies and damselflies, respectively. Although the karyotypes of most Odonata contain single pairs of 45S rDNA clusters, the position of NORs may differ in members of these suborders. Specifically, these sites are located on one of the largest autosomes in all studied members of Anisoptera but on m-chromosomes in many members of Zygoptera. Since the former pattern is considered to be ancestral for Odonata, the distribution of 45S rDNA sites found in members of Zygoptera is believed to be derived. Moreover, the latter pattern could have arisen in a common ancestor of the superfamilies Calopterygoidea and Coenagrionoidea [64]. On the other hand, FISH with an 18S rDNA probe revealed a single signal on a particular bivalent in the male meiotic karyotype of *Rhionaeschna planaltica* (Calvert) (Aeshnidae). However, detailed analysis identified this bivalent as neo-XY, with the neo-X chromosome apparently originated via fusion of the initial X chromosome and an m-chromosome [144].

**Polyneoptera (orthopteroid insects)** is a monophyletic group comprising 10 insect orders, which are predominantly characterized by monocentric chromosomes, with the exception of Dermaptera [4] and Zoraptera [146]. Data on the number and position of rDNA clusters for most of them are unavailable (Zoraptera, Dermaptera, Grylloblatodea, and Isoptera) or known for a few members, e.g., in the orders Plecoptera (stoneflies), Embioptera (webspinners), Mantodea (praying mantises), Blattodea (cockroaches and roaches), and Mantophasmatodea (heelwalkers). Specifically, in the stonefly *Skwala compacta* (McLachlan) (Perlodidae), 45S rDNA sites occupy the entire length of the shorter arms of both subtelocentric X and Y chromosomes, also stretching into the proximal parts of their longer arms [55]. In the webspinner *Embia* cf. *savignyi* Westwood (Embiidae), 45S rDNA clusters are also located on the shorter arms of a single pair of subtelocentric autosomes [147]. In the European mantis *Mantis religiosa* Linnaeus (Mantidae), for which the male karyotype is 2*n* = 24 + X_1_X_2_Y, silver staining revealed active NORs associated with the sex trivalent in meiosis [148]. A single 45S rDNA site was visualized on the X chromosome of the American cockroach *Periplaneta americana* (Linnaeus) [118]. However, in the Afrotropical subfamily Oxyhaloinae (Blaberidae), FISH detected NORs on two pairs of autosomes in the speckled cockroach *Nauphoeta cinerea* Olivier [149] and intraspecific variation of 18S rDNA loci in four other species [150]. Apart from these results, multiple NORs have been found on the chromosomes of the heelwalker *Karoophasma biedouwense* Klass et al. (Austrophasmatidae), i.e., a large rDNA cluster on the largest autosomal bivalent, together with smaller FISH signals on three other bivalents as well as on the X chromosome [42].

The 45S rDNA sites of six members of Phasmatodea (stick insects) were examined using FISH [70,118,151]. In four of them, belonging to the families Phasmatidae, Heteropterygidae, Lonchodidae, and Pseudophasmatidae, FISH signals occupy the entire shorter arm of the single large pair of metacentrics/submetacentrics, and additional subterminal signals were also found on a few other chromosomes [70]. However, an obvious heteromorphism of these sites was observed in *Phaenopharos khaoyaiensis* Zompro (Lonchodidae). Specifically, a single large submetacentric showed an obvious rDNA signal along its whole longer arm, whereas another homolog with a small dot-like signal was much shorter. In addition, the number and location of rDNA sites on homologous chromosomes dramatically vary among different species and individuals of the genus *Bacillus* Berthold (Bacillidae) [14]. Certain results demonstrate that co-localization of highly amplified rDNA and telomeric repeats on chromosomes is a shared ancestral trait for phasmids. Although the functional significance of this phenomenon is not entirely clear, it is suggested that, at least in *Bacillus*, co-localized repetitive sequences represent hotspots of chromosomal rearrangements that allow unequal crossovers ([151] and references therein).

Orthoptera (grasshoppers, locusts, and crickets) apparently represent one of the best studied insect orders in terms of the number and position of rDNA clusters ([17,38,76,77,85,87,93,118,130,131,152,153,154,155,156] etc.). In particular, these sites have been revealed on chromosomes of about 300 members of this taxon, which belong to both suborders, i.e., Ensifera and Caelifera [16]. In the former suborder (crickets and katydids), Tettigoniidae, with more than 160 studied species, remains the best studied family, as opposed to three members of Caelifera (grasshoppers and locusts), and Acrididae, Pamphagidae, and Romaleidae, with about 70, 40, and 10 examined species, respectively. Several other members of the families Gryllidae and Mogoplistidae (Ensifera) as well as Ommexechidae, Morabidae, Proscopiidae, and Pyrgomorphidae (Caelifera) were also studied in this respect.

Although one or two 45S rDNA clusters per haploid genome is most characteristic of Orthoptera, the highest known value of this parameter is 10, detected in *Abracris flavolineata* (De Geer), *Aeropus* (=*Gomphocerus*) *sibiricus* (Linnaeus), and *Eyprepocnemis plorans* [92,97,152]. The most frequent number of 5S rDNA sites ranges from one to three per haploid karyotype, but 12 such clusters were found in *Sphingonotus azurescens* (Rambur), *Chorthippus jacobsi* Harz, and *C. nevadensis* Pascual [130]. Interestingly, all of these insects with maximum numbers of rDNA sites belong to the family Acrididae. The wide occurrence of multiple clusters of ribosomal genes among members of Orthoptera is likely due to the much larger genome sizes in this order compared to other insects [157]. In turn, an increase in the copy numbers of 45S and 5S rRNA genes leads to loss of function by some of them, i.e., to their partial pseudogenization [135]. In Orthoptera, both 45S and 5S rDNA clusters mostly have either pericentromeric or interstitial locations, although subterminal sites of this kind were also found in several species [16]. Nevertheless, at least in some members of Acrididae, normal and pseudogenized 5S rDNA arrays are not co-localized [38].

**Paraneoptera (hemipteroid assemblage)** includes four orders with holokinetic chromosomes, although there are no records of rDNA clusters for the two of these groups, i.e., Phthiraptera and Thysanoptera. However, the number and position of rDNA sites have been studied in more than 200 species of the order Hemiptera (aphids, cicadas, planthoppers, true bugs, etc.), which is subdivided into four suborders. Among them, characteristics of rDNA clusters in the suborder Coleorrhyncha remain completely unknown. However, other suborders of Hemiptera are also unevenly studied in this respect. For example, of 50 species of Auchenorrhyncha (cicadas, leafhoppers, planthoppers etc.), members of three superfamilies, i.e., Cercopoidea, Cicadoidea, and Fulgoroidea, have been examined so far [16]. In the first superfamily, 45S rDNA clusters of 10 species of the family Cercopidae were studied together with eight members of the genus *Philaenus* Stål (Aphrophoridae) [67,158]. Although these *Philaenus* species are closely related, FISH revealed substantial differences in both the number and position of NORs. Specifically, diploid chromosome sets of different *Philaenus* species can carry two, three, or four 45S rDNA sites. Moreover, these clusters can be located on autosomes and/or sex chromosomes (X and/or Y) [67]. Similarly, five members of the genus *Magicicada* Davis (Cicadoidea, Cicadidae) also have different numbers of NORs, with four species having a single pair of large 45S rDNA clusters, whereas the fifth one has seven smaller clusters of this kind [159]. In addition, more than a dozen other members of Cicadoidea that belong to the families Aetalionidae, Cicadellidae, Membracidae, and Myerslopiidae have been studied [158,160,161]. Furthermore, 11 species of the planthopper family Issidae (Fulgoroidea) also show some restricted variation in the number and position of 45S rDNA sites [83]. In this family, the interstitial location of these clusters is widespread, whereas a few known cases of subterminal position of these sites are associated with chromosomal rearrangements involving 45S rDNA loci.

Up to now, 45S rDNA clusters have been studied in less than 20 members of the relatively large suborder Sternorrhyncha. Indeed, single species were examined in each of the two superfamilies, Aleyrodoidea and Coccoidea, although rDNA clusters in Phylloxeroidea were never studied. A single NOR was detected in the haploid male karyotype of the whitefly *Bemisia tabaci* (Gennadius) [162], and four 28S rDNA sites were frequently observed in the diploid chromosome set of the mealybug *Planococcus citri* (Risso), although up to six additional sites were occasionally detected [40].

On the other hand, in 12 aphid species of the family Aphididae (Aphidoidea), limited variation in the number of both 45S and 5S rDNA clusters was observed [20,53,118,163,164,165,166,167]. Specifically, all of these loci respectively occupy subterminal and interstitial positions, but their numbers in the diploid karyotypes of aphids vary from one to two and from one to four in 45S and 5S rDNA, respectively. Moreover, 45S rDNA clusters in this group are usually restricted to a single telomere of the X chromosome [20,53,118]. Substantial variation in the location of 45S rDNA sites was also discovered in Psyllidae and Aphalaridae, which both belong to the superfamily Psylloidea (jumping plant lice) [49]. In these two families, only five species have been studied to date, of which three possess subterminal 45S rDNA clusters. Although the remaining two species, *Rhinocola aceris* (Linnaeus) and *Baeopelma foersteri* (Flor), belong to Aphalaridae and Psyllidae, respectively, they both have lower chromosome numbers than the majority of members of Psylloidea (2*n*♂ = 11–15 vs. 21–25). Moreover, the interstitial location of 45S rDNA clusters makes these sites good markers of independent chromosomal fusion in these unrelated species [49].

The majority of members of Hemiptera with known numbers and positions of rDNA sites belong to the suborder Heteroptera (true bugs) [16]. However, this information is unavailable for four of the seven infraorders of true bugs, i.e., Dipsocoromorpha, Enicocephalomorpha, Gerromorpha, and Leptopodomorpha. On the other hand, important data on 45S and 5S rDNA clusters were obtained, e.g., for members of Pentatomomorpha [34,54,61,63,168,169] and Cimicomorpha. Specifically, more than 100 species and subspecies of the latter infraorder currently studied in this respect belong to the subfamilies Triatominae and Harpactorinae of the family Reduviidae. Among the 19 examined genera of this group, extraordinary variation in the number and position of 45S rDNA sites was revealed [22,65,66,68,78,126,170,171,172,173,174]. Although 45S rDNA clusters in members of Reduviidae are mostly subterminal [66,174], they can vary in both number and location on particular chromosomes. Specifically, the haploid karyotypes of these insects carry one to four NORs. In addition, 45S rDNA sites can be located on one to three autosomes and/or one to three sex chromosomes [22]. Certain variation in the number and location of NORs was also observed among more than 20 species of the lace bug family Tingidae [46,175,176,177,178]. Although two 45S rDNA clusters with interstitial locations, which apparently represent an ancestral character state for Tingidae, predominate in their diploid chromosome sets, these sites can also be subterminal, with their overall number increasing up to four. Moreover, several different patterns of chromosomal location of 18S rDNA sites have been detected in this family. Indeed, these clusters can be situated either on a pair of autosomes, one or two sex chromosomes, or on an autosomal pair and the X chromosome [175]. In Cimicomorpha, a few other species that belong to the families Cimicidae, Nabidae, and Miridae usually also have two NORs per diploid karyotype [47,179,180,181]. In the water bug infraorder Nepomorpha, about 10 members of the genus *Belostoma* Latreille (Belostomatidae) [182,183,184] as well as two species of the families Nepidae and Naucoridae [185,186] have been studied. The diploid karyotypes of these groups typically contain single pairs of 45S rDNA clusters, although two *Belostoma* species have four such sites. In a few dozen species of Pentatomomorpha, rDNA clusters have also been examined. Among these insects, rDNA sites were studied in more than 20 members of Coreidae and in a similar number of Pentatomidae. The distribution of 45S rDNA sites is more or less uniform in various species of both families, i.e., there are two subterminal NORs per diploid karyotype [31,34,61,63,187,188,189,190]. However, the distribution of 5S rDNA in these groups is substantially more diverse. Specifically, diploid chromosome sets of most members of Coreidae carry two subterminal 5S rDNA clusters, although they can either be located on sex chromosomes or shift to an interstitial position in a few unrelated species. Moreover, 5S rDNA in *Chariesterus armatus* (Thunberg) does not form compact clusters but is spread throughout the whole karyotype [31]. In addition, some 5S rDNA arrays in this species are apparently pseudogenized, as is often the case in insects with strongly increased numbers of rDNA sites, such as in some members of Orthoptera (see above). Members of Pentatomidae share the same overall distribution pattern of rDNA clusters with those of Coreidae, with considerable variation in the number of 5S rDNA clusters. Among other Pentatomomorpha families, a few species in Rhopalidae, Lygaeidae, Scutelleridae, Pyrrhocoridae, and Largidae were also studied [54,61,63,118,168,169,189,191,192]. In these groups, 45S rDNA sites demonstrate characteristics similar to those in Coreidae and Pentatomidae.

In the order Psocoptera (barklice and booklice), NORs have been studied in eight members of five families, including Caeciliusidae, Stenopsocidae, Peripsocidae, Philotarsidae, and Psocidae [45]. All examined species were characterized by two subterminal 45S rDNA clusters per diploid karyotype.

**Holometabola (insects with full metamorphosis)** is an enormous group that harbors eleven insect orders, including a few largest ones, or the so-called “big four”, i.e., Coleoptera, Hymenoptera, Lepidoptera, and Diptera [1]. In these orders, various characteristics of NORs have already been studied in several hundred species. For example, rDNA clusters have been examined in about 180 members of Hymenoptera (sawflies, parasitoids, wasps, ants, bees, etc.). In turn, these insects belong to both suborders, Symphyta and Apocrita, or lower and higher Hymenoptera, respectively, although many available data on the number and position of their rDNA clusters are not currently included in the existing database [16]. Nevertheless, NORs in Hymenoptera are usually represented by a single 45S rDNA locus, either pericentromeric or subterminal, per haploid karyotype [19]. For example, in the superfamily Tenthredinoidea (Symphyta), these sites were studied in four members of the families Diprionidae and Tenthredinidae (see, e.g., [193]). In both species of Diprionidae, *Diprion pini* (Linnaeus) and *Neodiprion abietis* (Harris), a single subterminal NOR was found on an acrocentric chromosome [194,195]. However, all 45S rDNA clusters revealed in another three members of the family Tenthredinidae, *Nematus ribesii* (Scopoli), *Tenthredo mesomela* Linnaeus, and *Athalia rosae* (Linnaeus), are pericentromeric. Moreover, the karyotypes of *N. ribesii* and *T. mesomela* also harbor a single NOR, whereas that of *A. rosae* carries four such sites [118,196,197].

The suborder Apocrita, which, in turn, is subdivided into two traditional large groups, Parasitica and Aculeata, is substantially better studied in terms of the number and location of rDNA clusters. However, the overwhelming majority of species with known characteristics of these clusters belong to Aculeata, whereas less than 30 members of the former group have been studied in this respect. Specifically, in the superfamily Ichneumonoidea, two members of the family Braconidae, which have divergent chromosome numbers, *Cotesia congregata* (Say) with *n* = 10 and *Diachasmimorpha longicaudata* (Ashmead) with *n* = 20, also show strong differences in the number of 45S rDNA clusters, i.e., one and six, respectively [198,199]. Interestingly, the only studied member of the sister family Ichnemonidae, *Ichneumon amphibolus* Kriechbaumer, with 2*n* = 24, has three pairs of NORs, thus demonstrating a somewhat intermediate number of 45S rDNA sites [44]. In a few species of the superfamily Cynipoidea, 45S rDNA clusters have also been examined [42,200]. Although all haploid karyotypes of these insects contain single NORs, their positions can differ across different species. Indeed, *Ganaspis xanthopoda* (Ashmead) and *Leptopilina boulardi* (Barbotin et al.) (Figitidae) share the same chromosome number, *n* = 9, and pericentromeric 45S rDNA clusters, but these sites are located on a largest metacentric and a small acrocentric chromosome, respectively. On the other hand, in two related species of *Leptopilina* Förster, i.e., *L. heterotoma* (Thomson) and *L. victoriae* Nordlander, subterminal NORs appear to be situated on the shorter arm of the same subtelocentric chromosome [200]. This location is similar to that found in the only examined member of the large family Cynipidae, *Diplolepis rosae* (Linnaeus) [44]. Similarly, 45S rDNA clusters have been studied in less than 20 species of the vast superfamily Chalcidoidea [201], including members of Aphelinidae, Eulophidae, Eurytomidae, Pteromalidae, Torymidae, and Trichogrammatidae [44,57,202,203,204,205,206,207,208,209,210]. The haploid karyotypes of these parasitoids usually harbor one or two pericentromeric NORs, as, for example, can be observed in three *Eurytoma* Illiger species, but in *Trichogramma kaykai* Pinto et Stouthamer, both of these sites are subterminal [44,207]. Moreover, an additional 45S rDNA cluster is located on the B chromosome in the latter species. In an individual of *Torymus bedeguaris* (Linnaeus), with 2*n* = 13, which appears to be trisomic for the smallest acrocentric (see [211]), NORs are detected on all three copies of this chromosome [44].

Up to now, 45S rDNA sites have been studied in about 160 species of aculeate Hymenoptera, with most members of this group belonging to the superfamilies Formicoidea and Apoidea [19]. Nevertheless, the number and location of NORs are also known for more than 20 species of Vespidae (Vespoidea) [19,62,212,213,214,215,216]. Although a single 45S rDNA cluster per haploid karyotype apparently represents the ancestral character state for this family, and perhaps for Hymenoptera in general (see above), the highest number of NORs known in insects, i.e., 15, was also found in a member of this group, *Synoeca surinama* (Linnaeus) [62]. In addition to Vespoidea, 45S rDNA clusters were studied in two families of Apoidea, i.e., Crabronidae and Apidae. Among members of Crabronidae, NORs have been examined only in four members of the genus *Trypoxylon* Latreille [88]. Different *Trypoxylon* species appear to have either one or two pericentromeric 45S rDNA clusters per haploid karyotype. In addition, the number and position of NORs on chromosomes of more than 40 members of Apidae are known up to now [19,124,205,217,218,219,220,221,222,223,224,225,226,227,228,229,230]. Although the number and position of 45S rDNA clusters were previously studied in *Apis mellifera* Linnaeus and a few other bees, the overwhelming majority of examined species belong to the large genus *Melipona* Illiger [221,222,223,230]. All diploid karyotypes of this group harbor a single NOR; however, this NOR tends to be either pericentromeric or subterminal in species with low and high amount of heterochromatin, respectively. Moreover, the chromosome pair carrying NORs in the diploid set is often heteromorphic [226,230].

Ants, or the family Formicidae (Formicoidea), by far represent the most studied group of Hymenoptera in terms of 45S rDNA clusters, with about a hundred examined species [19,21,23,39,89,94,122,231,232,233,234,235,236,237,238,239,240,241,242,243,244,245,246,247,248,249,250,251,252]. Nevertheless, the number and distribution of NORs across ant karyotypes are generally consistent with the abovementioned patterns. Specifically, a single 45S rDNA cluster per haploid set is characteristic of most species of this group and is therefore considered to be an ancestral character state for this family [21]. In addition, these loci are mostly pericentromeric or subterminal. However, the number of NORs can substantially vary among closely related species. For example, the diploid karyotypes of different members of the genus *Myrmecia* harbor from two to 19 45S rDNA clusters [39].

Apart from Hymenoptera, other orders of Holometabola are united within the latter group into several higher taxa. For instance, Neuropterida, which is sometimes considered to be a superorder, contains three orders, i.e., Raphidioptera, Megaloptera, and Neuroptera. Among these groups, 45S rDNA clusters have been studied only in four members of the latter order that belong to the families Chrysopidae and Myrmeleontidae [48,118]. In Myrmeleontidae, the diploid karyotypes of *Palpares libelluloides* (Linnaeus) of the subfamily Palparinae, *Acanthaclisis occitanica* (Villers) (Acanthaclisinae) and *Distoleon tetragrammicus* (Fabricius) (Nemoleontinae), harbor a pair of large autosomes that bears an NOR on each homolog, with an additional 45S rDNA site on one of the sex chromosomes, presumably on X, in the latter two species. Interestingly, they both have significantly lower chromosome numbers, 2*n* = 16 + XY and 2*n* = 14 + XY, respectively, compared to the first species, with 2*n* = 24 + XY [48]. In addition, the meiotic chromosome set of *Chrysoperla carnea* (Stephens) (Chrysopidae), with 2*n* = 10 + XY, contains a single NOR on the XY bivalent [118].

In the superorder Coleopterida, which contains the orders Coleoptera (beetles) and Strepsiptera (twisted-wing parasites), there are no data on the number and distribution of NORs in Strepsiptera. On the contrary, around 250 species of Coleoptera have been studied in this respect up to now. Specifically, among species that belong to the suborder Adephaga, rDNA clusters have been examined in about 50 members of the family Cicindelidae [253,254,255,256,257,258,259,260,261]. In this group, NORs are relatively diverse in both their number and location, varying from two to eight per diploid karyotype. In addition, 45S rDNA clusters can occupy subterminal, interstitial, or pericentromeric positions on autosomes as well as on X or Y chromosomes [254,259]. On the other hand, these clusters have been studied in approximately 60 species and subspecies of the large family Carabidae [72,129,262]. Although NORs in these beetles usually occupy subterminal positions on chromosomes, the number and size of these sites substantially vary within certain taxa. For example, the diploid karyotypes of the genus *Zabrus* Clairville harbor from two to twelve 45S rDNA clusters [72]. Moreover, the apparent size of NORs in Carabidae varies from dot-like signals to those covering entire chromosome arms [72,262]. In *Bembidion lividulum* Casey, preliminary sequencing of the 45S rDNA revealed strong inflation of the copy number of the 28S rRNA gene, as opposed to 18S. It is therefore not surprising that FISH with the latter probe showed two compact clusters on a particular chromosome pair, whereas FISH with a 28S rDNA probe demonstrated diffuse signals across all chromosomes [129] (also see above).

For many species of the suborder Polyphaga, there are no data on the number and location of rDNA sites. Nevertheless, the superfamily Scarabaeoidea remains the best-known group in Polyphaga in this respect, with more than 100 studied members ([41,69,263,264] etc.). In fact, almost all of these species belong to the family Scarabaeidae [41,69,114,118,263,264,265,266,267,268,269,270]. Moreover, both 45S and 5S rDNA clusters were studied in many members of the latter group. The number and location of these sites proved to be substantially variable. In particular, one to sixteen and one to seven clusters of 45S and 5S rDNA, respectively, were found in the diploid karyotypes of different members of Scarabaeidae [41,69,263,264,267,270]. While the 5S rDNA sites are always located near the centromere, the position of 45S rDNA clusters can be either pericentromeric, subterminal, or interstitial. In the European cockchafer *Melolontha melolontha* (Linnaeus), a particular chromosome carrying the so-called fragile NOR is also prone to recurrent fission and frequent chromatid exchange with other chromosomes [114]. In addition, NORs in a few species from other families of Scarabaeoidea, i.e., Geotrupidae, Lucanidae, and Passalidae, were also studied [263,271,272,273,274]. Among several examined members of Coccinelloidea and Tenebrionoidea, the number and position of 45S rDNA clusters in six species that belong to the families Coccinellidae, Meloidae, and Tenebrionidae are known [37,43,205,275,276]. Interestingly, the diploid karyotype of *Hycleus scutellatus* (Rosenhauer) (Meloidae), with 2*n* = 20, carries 12 subterminal NORs [43]. Since 45S rDNA clusters are located on most autosomes, this is the highest number of NORs found in Polyphaga in general.

In the suborder Phytophaga, more than 20 members of the superfamilies Elateroidea, Chrysomeloidea, and Curculionoidea, as well as of their nominative families, Elateridae, Chrysomelidae, and Curculionidae, were studied [225,277,278,279,280]. In these groups, 45S rDNA clusters are usually subterminal and interstitial, except in members of Curculionidae, in which pericentromeric sites of this kind prevail. The number of NORs in the examined members of Chrysomelidae can vary from two to six per diploid karyotype.

The large superorder Amphiesmenoptera includes insects of two sister orders, Trichoptera (caddisflies) and Lepidoptera (butterflies and moths), which both have holokinetic chromosomes (see, e.g., [281]). In fact, rDNA sites have been studied in the only caddisfly species, *Glyphotaelius pellucidus* (Retzius) (Limnephilidae). Its diploid karyotype contains single pairs of 45S and 5S rDNA clusters with subterminal locations [32]. This position of 45S rDNA is also characteristic of many members of the order Lepidoptera and is therefore currently considered to be an ancestral character state for this group [18,32,128,281,282,283,284]. At present, the number and position of rDNA clusters are known for about 50 species of Lepidoptera, but chromosomes of a few members of this group carry interstitial 45S rDNA sites [18,32]. In addition, the number of NORs can increase up to two, seven, and eleven in *Nymphalis xanthomelas* Denis et Schiffermüller, *Aglais urticae* (Linnaeus), and *Inachis io* (Linnaeus) (Nymphalidae), respectively [18,32], which suggests that the elevated number of rDNA clusters is a characteristic feature of the latter family. In two closely related species of the genus *Abraxas* Leach (Geometridae), *A. grossulariata* (Linnaeus) and *A. sylvata* (Scopoli), the main rDNA sites are detected on both Z and W chromosomes, but the karyotype of the former species carries another autosomal NOR. Moreover, the main rDNA cluster is located at the very end of the bivalent in *A. grossulariata* but it is clearly subterminal in *A. sylvata* [284]. Interestingly, all three NORs found in *Taleporia tubulosa* (Retzius) (Psychidae) are confined to the same bivalent [32]. As far as 5S rDNA clusters are concerned, their distribution has been studied in just a few species of Lepidoptera. In all of them, a particular bivalent carries a single subterminal site of 5S rDNA [32].

The large superorder Antliophora harbors insects that belong to three orders, i.e., Mecoptera, Diptera, and Siphonaptera. However, the number and position of rDNA sites have been examined only in members of the two former taxa. In Mecoptera (scorpionflies or hanging flies), only the only species *Panorpa vulgaris* Imhoff et Labram (Panorpidae) has been studied. In the male karyotype of *P. vulgaris*, FISH with an 18S rDNA probe yielded four positive signals, i.e., apparent NORs [118].

To date, rDNA clusters have been examined in about 80 members of the order Diptera (midges, mosquitoes and flies). Although the position of these clusters in Diptera can sometimes be traced more precisely due to presence of giant polytene chromosomes in salivary glands ([285] etc.), the locations of these sites in many dipterans were visualized on mitotic metaphase chromosomes (see below). The order Diptera is often subdivided into two traditional groups, Nematocera and Brachycera, and certain members of the former one, i.e., the families Culicidae and Chironomidae, remain the best studied in terms of rDNA clusters. Specifically, NORs were examined in more than 20 species of Culicidae [96,127,286,287,288,289]. For example, 45S rDNA clusters are detected on the heterochromatic arms of the X chromosome and sometimes also of the Y chromosome in the genus *Anopheles* Meigen [127,286,287,288]. In the diploid karyotype of a particular strain of *Aedes aegypti*, FISH revealed an additional unpaired NOR that appeared to be AT-rich, since it was brightly stained with DAPI but not with CMA_3_ [96]. In the family Chironomidae, rDNA clusters were examined in approximately the same number of species [285,290,291]. For instance, three and six 45S and 5S rDNA sites were respectively visualized on chromosomes of the haploid karyotype of *Glyptotendipes barbipes* (Staeger) [290]. Moreover, a modern study of 45S rDNA distribution discovered considerable variation in the number and position of these clusters in the genus *Chironomus* Meigen [286]. Although a single site per haploid set represents the prevailing character state in this group, the number of NORs sometimes increases to six, and they can be located on any chromosome arm in different species. In addition to Culicidae and Chironomidae, rDNA clusters were examined in two other members of Nematocera, which belong to the genus *Bradysia* Winnertz (Sciaridae), namely, *B. hygida* Sauaia et Alves and *B. tilicola* (Loew) (=*Sciara coprophila* Lintner) [292,293,294,295]. For example, AgNOR staining and FISH revealed single 45S and 5S rDNA sites on two different autosomes in the former species [293,294,295]. On the contrary, silver impregnation visualized NOR in the proximal heterochromatic segment of the X chromosome of *B. tilicola* [292].

In Brachycera, the number and position of rDNA clusters have also been studied in a few families. For instance, these sites were examined in three members of the genus *Pseudacteon* Coquillett (Phoridae) [296]. In all studied species with 2*n* = 6, FISH with an 18S rDNA probe revealed a single pericentromeric NOR on the second chromosome pair. Another comparative study of this kind involved seven members of the genus *Glossina* Wiedemann (Glossinidae) [297]. In most species, a single interstitial 45S rDNA cluster is detected on a particular pair of autosomes, but in *G. pallidipes* (Austen) and *G. palpalis* (Robineau-Desvoidy), the Y chromosomes carry an additional NOR. In two members of the genus *Lucilia* Robineau-Desvoidy (Calliphoridae), namely, *L. cluvia* (Walker) and *L. sericata* (Meigen), 45S rDNA sites were detected using both AgNOR staining and FISH [298]. Although NORs in these species are situated on both X and Y chromosomes, these chromosomes strongly differ in size and structure between *L. sericata* and *L. cluvia*, and the positions of these sites therefore differ as well [298]. The haploid karyotype of another member of this family, *Protocalliphora falcozi* Séguy, appears to carry a single 45S rDNA cluster [299]. In a particular strain of *Anastrepha fraterculus* (Wiedemann) (Tephritidae), a species with multiple sex chromosomes that actually represents a complex of cryptic taxa, subterminal and pericentromeric NORs were detected on two sex chromosomes, X_1_ and Y_5_ [300,301]. Similarly, FISH with an 18S rDNA probe revealed signals on the X and Y chromosomes in another tephritid, *Bactrocera oleae* (Rossi) [302]. In each of the two other members of this family, *Rhagoletis pomonella* (Walsh) and *Ceratitis capitata* (Wiedemann), NORs were visualized on a particular autosome pair; however, in the latter species, additional NORs were detected on chromosomes X_2_ and Y_5_ [301,303]. Finally, clusters of ribosomal genes were studied in more than 20 species of *Drosophila* (Drosophilidae) [51,133,304,305,306,307]. In this genus, 45S rDNA sites are usually represented by single clusters on both sex chromosomes. Interstitial and pericentromeric positions on the X and Y chromosomes, respectively, are currently considered to be ancestral character states for several species groups of Drosophila. The Y chromosome can either acquire an additional 45S rDNA cluster or completely lack these sites in particular members of this genus [51,133]. On the other hand, the X chromosome can also lose its 45S rDNA site, which then reappears on a particular autosome [51]. In both *D. hydei* Sturtevant and *D. melanogaster*, single 5S rDNA clusters apparently have an interstitial location on the second chromosome [305,307].

## 8. Conclusions

Currently, clusters of 45S and 5S rDNA have been studied in about 1000 and 100 species of the class Insecta, respectively. Although the number of insect species with known 45S rDNA clusters, or NORs, constitutes less than 0.1 percent of the described members of this enormous group, certain conclusions can already be drawn. Since haploid karyotypes with single 45S and 5S rDNA clusters predominate in both basal and derived insect groups, this character state is apparently ancestral for the class Insecta in general. Nevertheless, the number, chromosomal location, and other characteristics of both 45S and 5S rDNA sites substantially vary across different species, and sometimes even within the same species. There are important molecular mechanisms that either maintain these parameters or alter them on the short-term and/or long-term scale. We are currently trying to pinpoint and explain the abovementioned mechanisms, but further observations and experimental data are needed to confirm or refute these hypotheses.

## Data Availability

No new data were created or analyzed in this study. Data sharing is not applicable to this article.
